# Factors influencing patient adherence with diabetic eye screening in rural communities: A qualitative study

**DOI:** 10.1371/journal.pone.0206742

**Published:** 2018-11-02

**Authors:** Yao Liu, Nicholas J. Zupan, Olayinka O. Shiyanbola, Rebecca Swearingen, Julia N. Carlson, Nora A. Jacobson, Jane E. Mahoney, Ronald Klein, Timothy D. Bjelland, Maureen A. Smith

**Affiliations:** 1 Department of Ophthalmology and Visual Sciences, University of Wisconsin School of Medicine and Public Health, Madison, Wisconsin, United States of America; 2 Health Innovation Program, University of Wisconsin School of Medicine and Public Health, Madison, Wisconsin, United States of America; 3 Department of Social and Administrative Sciences, University of Wisconsin School of Pharmacy, Madison, Wisconsin, United States of America; 4 Institute for Clinical and Translational Research, University of Wisconsin School of Medicine and Public Health, Madison, Wisconsin, United States of America; 5 Department of Medicine, University of Wisconsin School of Medicine and Public Health, Madison, Wisconsin, United States of America; 6 Mile Bluff Medical Center, Mauston, Wisconsin, United States of America; 7 Department of Population Health Sciences, University of Wisconsin School of Medicine and Public Health, Madison, Wisconsin, United States of America; 8 Department of Family Medicine and Community Health, University of Wisconsin School of Medicine and Public Health, Madison, Wisconsin, United States of America; University Lyon 1 Faculty of Dental Medicine, FRANCE

## Abstract

**Objective:**

Diabetic retinopathy remains the leading cause of blindness among working-age U.S. adults largely due to low screening rates. Rural populations face particularly greater challenges to screening because they are older, poorer, less insured, and less likely to receive guideline-concordant care than those in urban areas. Current patient education efforts may not fully address multiple barriers to screening faced by rural patients. We sought to characterize contextual factors affecting rural patient adherence with diabetic eye screening guidelines.

**Research design and methods:**

We conducted semi-structured interviews with 29 participants (20 adult patients with type 2 diabetes and 9 primary care providers) in a rural, multi-payer health system. Both inductive and directed content analysis were performed.

**Results:**

Factors influencing rural patient adherence with diabetic eye screening were categorized as environmental, social, and individual using the Ecological Model of Health. Major themes included limited access to and infrequent use of healthcare, long travel distances to obtain care, poverty and financial tradeoffs, trusting relationships with healthcare providers, family members’ struggles with diabetes, anxiety about diabetes complications, and the burden of diabetes management.

**Conclusions:**

Significant barriers exist for rural patients that affect their ability to adhere with yearly diabetic eye screening. Many studies emphasize patient education to increase adherence, but current patient education strategies fail to address major environmental, social, and individual barriers. Addressing these factors, leveraging patient trust in their healthcare providers, and strategies targeted specifically to environmental barriers such as long travel distances (e.g. teleophthalmology) may fill crucial gaps in diabetic eye screening in rural communities.

## Introduction

Diabetic retinopathy affects an estimated 4.2 million Americans and is the most common cause of blindness in working-age U.S. adults [1, 2]. Early diagnosis and treatment decrease the risk of severe vision loss by 90%, but fewer than half of the 29.1 million Americans with diabetes receive yearly eye screening in accordance with recommended guidelines [3–5]. Rural populations face particularly greater challenges to obtaining diabetic eye screening because they are older, poorer, less insured, and less likely to receive guideline-concordant care, while also experiencing more chronic disease—including more severe diabetic retinopathy—than those in urban areas [6–8].

Patient adherence with yearly screening guidelines is critical to prevent blindness from diabetic eye disease. Several studies recommend patient education interventions to increase adherence with diabetic eye screening [9–12]. Although education is important, screening rates in the U.S. have stagnated at 50% despite patient and provider education [13–16]. Furthermore, patient education fails to address multiple additional barriers experienced by patients living in rural communities, such as limited transportation and access to health care services [17].

Understanding contextual factors affecting patient adherence may allow for the design of successful interventions to increase diabetic eye screening in rural health settings. Qualitative methods are useful in health services research to explore these types of complex phenomena [18]. We performed individual interviews, analyzed using both inductive and directed content analysis, to characterize contextual factors affecting patient adherence with yearly diabetic eye screening guidelines in a rural, multi-payer health system.

## Research design and methods

### Research setting

We performed semi-structured interviews with patients with diabetes and primary care providers (PCPs) at Mile Bluff Medical Center. Mile Bluff is a rural, multi-payer health system in Juneau County, Wisconsin. Juneau County’s population (n = 26,274, density = 34.8/square mile) [19] is 83.5% rural and is ranked among the lowest quartile of counties in Wisconsin according to national health metrics [20]. Compared to statewide averages, the population of Juneau County has a 17% lower median household income and 24% fewer residents with some college education, as well as a 33% higher prevalence of diabetes, 20% higher prevalence of obesity, and a 28% greater population over age 65 years [20].

### Interviews

We developed our semi-structured interview guides using the Chronic Care Model, a practical framework for improving chronic disease management and guideline-concordant diabetes care [21, 22]. While interview guides were initially developed to capture patients’ and providers’ perspectives on clinical barriers and facilitators for diabetic eye screening, participants also described important contextual factors influencing rural patients’ adherence with screening guidelines that we sought to capture and categorize in this study using a second model, the Ecological Model of Health [23].

Following a literature search on barriers and facilitators to diabetic eye screening, the patient interview guide (S1 Text. Patient Interview Guide) was tested and further refined with input from the University of Wisconsin Community Advisors on Research Design and Strategies (CARDS), a group of lay community members trained to review patient research materials. The PCP interview guide (S2 Text. Primary Care Provider Interview Guide) was tested and further refined with the input of PCPs from the University of Wisconsin Primary Care Academics to Transform Healthcare (PATH). Members of the University of Wisconsin Institute for Clinical and Translational Research-Community Academic Partnership (UW ICTR-CAP) Qualitative Research Group also reviewed and provided feedback on both interview guides.

Semi-structured, individual interviews to understand patient and PCP perspectives on diabetic eye screening were performed between July 2016 and April 2017. All interviews were conducted by a research specialist (R.S.) with training in qualitative research and certification as a nursing assistant. Patient interviews were performed in-person (30–45 minutes) at a local library. PCP interviews were conducted over the phone (15–30 minutes) to accommodate their busy clinic schedules. After the interview, patients received $30 and PCPs received $50 compensation for their time. Interviews were audiotaped and transcribed verbatim with all personal identifiers redacted.

### Study sample

The sample of 20 adult patients with diabetes and 9 PCPs (Table 1) was drawn from patients and providers at Mile Bluff Medical Center. Adult patients (18 year or older) with a diagnosis of diabetes who were recruited had expressed interest in participating in a research study when previously contacted in a quality improvement telephone survey on diabetic eye screening. All patients had a PCP from Mile Bluff Family or Internal Medicine. PCPs were recruited during a provider staff meeting with purposeful recruitment of providers having a range of training backgrounds (e.g. MD/DO, PA-C, etc.) reflective of their representation at Mile Bluff. Sample sizes for both patients and PCPs were sufficient to reach informational redundancy in which no new information was obtained from additional interviews [24].

**Table 1:** Patient and primary care provider demographics.

Participant Characteristics	Median or Percentage
Patients (n = 20)	
Age	67 years (range: 46–86 years)
Male	55%
Ethnicity (self-reported)	
White, non-Hispanic	100%
Diagnosis of Type-2 Diabetes	100%
Duration of diabetes	
<5 years	40%
5–19 years	30%
20+ years	30%
Highest Level of Education	
College graduate	10%
Some college/tech school	30%
High school graduate or GED	35%
Some high school	15%
Grade 8 or less	10%
Health literacy (Single Item Literacy Screener)	
High	85%
Moderate	10%
Low	5%
Primary Care Providers (n = 9)	
Male	77.8%
Training Background	
MD/DO	44.4%
PA-C	33.3%
DNP	11.1%
RN	11.1%
Years in Practice	
>10 years	77.8%
5–10 years	0%
0–5 years	22.2%

### Data analysis

The transcripts were first analyzed using inductive analysis. Open coding of the first 5 transcripts was performed independently by 3 members of the research team (R.S.-research specialist and certified nursing assistant, N.Z.-research specialist with a Master of Public Health degree, and Y.L.-clinical ophthalmologist and principal investigator). These research team members then met together with N.J.-Ph.D. qualitative methodologist to review these codes and agree upon a coding framework. A second-coding cycle was then performed by 1 research team member (N.Z.) to fit codes into higher-order categories. Consistency was ensured by dual-coding of every 5^th^ transcript by the principal investigator (Y.L.). Codes were iteratively reviewed and further refined by the research team. The interview data and coding methods were also reviewed by members of the UW ICTR-CAP Qualitative Research Group. Data management was facilitated using NVivo software, Version 11.4.1 (QSR International, Melbourne, Australia).

Member checking to validate our data analysis was performed with a subset of interview participants (n = 9 patients, n = 6 PCPs) at two separate patient and provider stakeholder group meetings organized as part of a quality improvement initiative to increase diabetic eye screening at Mile Bluff [25]. Our interpretation of the interview data was judged to be accurate and complete by participating patients and providers. We followed the Consolidated criteria for reporting qualitative research (COREQ) checklist in the report of our study [26].

### Ethics/Institutional Review Board Review

The University of Wisconsin School of Medicine and Public Health Human Subjects Institutional Review Board determined that this interview research met criteria for exemption. The interviewer obtained and documented verbal consent from all participants as approved by the above Institutional Review Board for this interview research.

## Results

All patients were rural, white adults diagnosed with type 2 diabetes (Table 1). Most (85%) self-reported high health literacy in response to the Single Item Literacy Screener [27]. PCPs came from a variety of training backgrounds and were predominantly male (77.8%). Most PCPs had been in practice for over 10 years (77.8%).

### A Model for understanding factors influencing patient adherence

**Fig 1:**
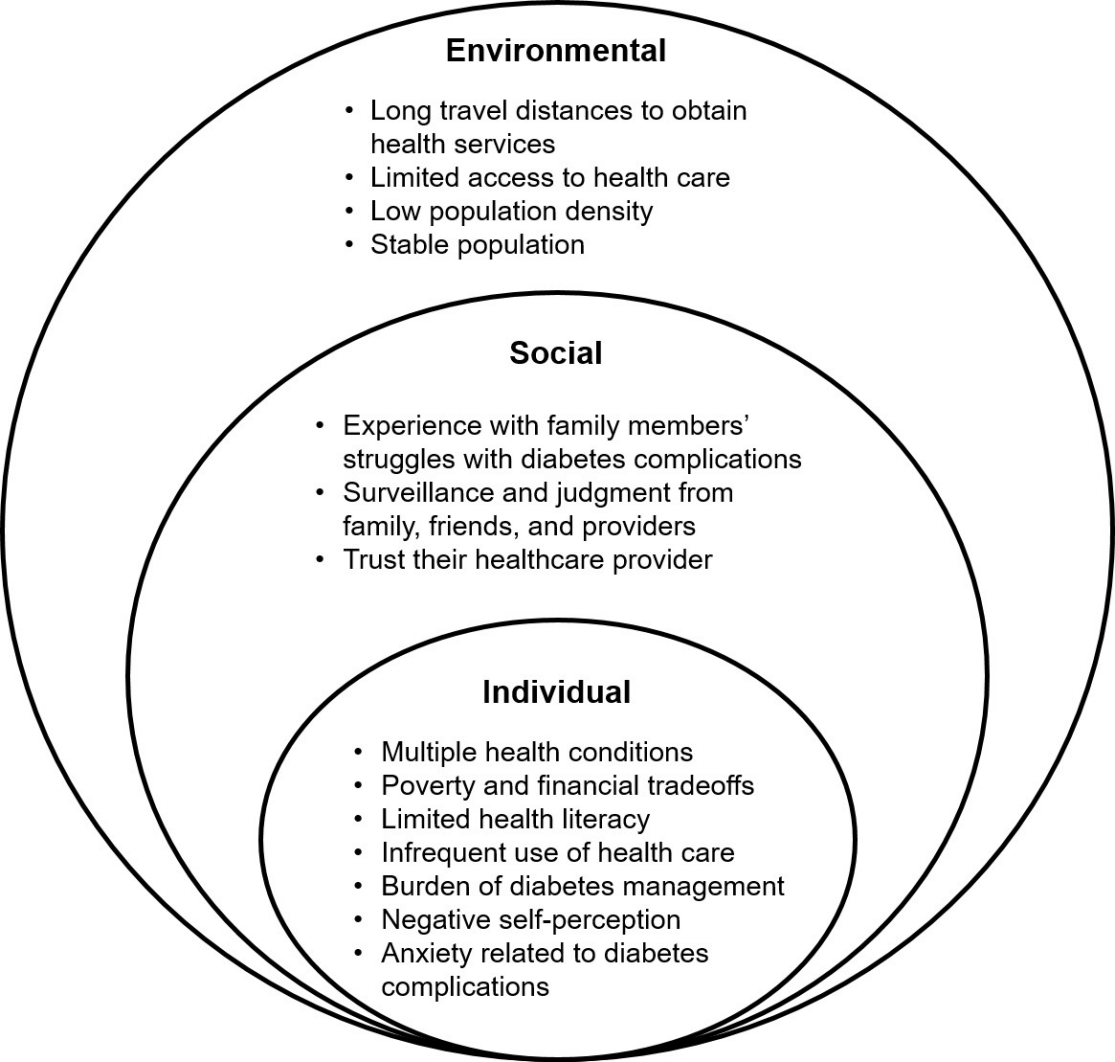
Factors influencing patient adherence with diabetic eye screening in rural communities (based on the Ecological Model of Health [23]).

We categorized themes identified from participant interviews using the Ecological Model of Health [23], including environmental, social, and individual factors (Fig 1). We chose the Ecological Model of Health to demonstrate the interrelationships between these factors relevant to rural patients’ adherence to diabetic eye screening.

### Environmental factors

Participants described several environmental factors related to obtaining diabetic eye screening in a rural community (Table 2). Patients frequently travel long distances to obtain health services and have limited access to health care. In addition to lengthy travel times and transportation barriers, participants also noted that health care providers had limited availability, particularly for specialty care. Thus, these structural barriers encouraged the utilization of healthcare on an “as-needed” basis, usually for urgent or emergency care, and the expectation of long wait times to obtain primary care appointments. Seeking ongoing healthcare for additional preventive services, especially from a specialist (e.g. eye doctors providing in-person diabetic eye screening) was often a lower priority and therefore less commonly performed due to these environmental factors.

**Table 2:** Environmental factors.

Theme	Quotes
Long travel distances to obtain health services	Interviewer: How far is the [eye clinic] from where you live? Patient: Between 65 and 70 miles. (PT #9) I have a lot of people from out of the area. I have people that drive 40–50 miles to come see us. (PCP #4)
Limited access to health care	We’re a rural community … We are kind of used to this… If you got an emergency, go to the emergency room, but you call your doctor [less often] and you get in when you can. (PT #13) My husband, he’s gonna be 87 in next month. He doesn’t like to drive anymore. And I do mostly all the driving now so it’s hard [to get to appointments]. (PT #15)
Low population density	And I live out in the wilderness, you know, out in the country. So, I don’t have nobody. I’m here living here permanently; 21 years and I know a handful of people. And that’s it… in 21 years. (PT #8)
Stable population	I’ve worked here 26 years, I kind of know how everything [goes] around here with healthcare. (PT #20)

Interestingly, patients did not necessarily consider these environmental factors to be negative, but rather an accepted part of living in a rural community. Furthermore, participants reported that the low population density was a positive factor that contributed towards attracting them to live there long-term. While some had lived in this community for most of their lives, others moved to the area post-retirement because they had previously enjoyed vacationing in the area for hunting and fishing. Many participants expressed pride that their rural community sustained a stable, welcoming population where most residents chose to live for several decades.

### Social factors

We identified multiple factors related to interpersonal relationships affecting rural patients’ motivation to obtain diabetic eye screening (Table 3). These included patients’ relationships with family, friends, and healthcare providers. Several patients reported having multiple family members with diabetes complications that provided firsthand knowledge of the severe consequences of poor diabetes control including amputation, renal failure, and vision loss. These experiences caused significant fear and anguish regarding the suffering of family members and the struggles patients personally experienced with diabetes management. In some cases, this fear and anguish motivated patients to seek regular diabetic eye screening in adherence with yearly screening guidelines to prevent these outcomes. However, participants also reported that seeing family members with severe diabetes complications sometimes led patients to avoid diabetic eye screening and other preventive health services due to the fear of receiving bad news about their health.

**Table 3:** Social factors.

Theme	Quotes
Experience with family members’ struggles with diabetes complications	So, I’m really working at it ‘cuz I know how diabetes is on your body… I watched my mom, she lost part of her eyesight, she lost toes… she was on kidney dialysis at the end. (PT #18) We call diabetes the ‘family curse.’ Every family reunion you’d see people with ‘hacked off limbs’ … (PT #20)
Surveillance and judgment from family, friends, and providers	People always ask me, ‘Well how can you have a candy bar?’ (PT #6) My daughter tells me every day. Mom, you’ve got to get off the couch, you got to walk. (PT #8) If they are not motivated… that’s of course the bane of all diabetes, right? People just aren’t motivated to deal with it. (PCP #2)
Trust in their healthcare provider	He’s a very good doctor and you know, I’m, I’m hoping he’ll never retire. (PT #2) I just do what my doctor tells me, basically. (PT #13)

In addition, patients described negative judgments from family, friends, and healthcare providers regarding their ability to adhere with diabetes management guidelines such as diabetic eye screening. Patients reported frustration that their daily dietary choices and exercise routines seemed to be closely monitored by others and frequently expressed a sense of being patronized or judged negatively when their behaviors did not follow guidelines. This atmosphere of monitoring and judgment created a sense of social stigmatization among patients that contributed to lowered self-esteem and to internalized beliefs that they lacked self-efficacy to adhere consistently with diabetes guidelines, including diabetic eye screening.

However, patients also noted that they had trusting, long-term relationships with their healthcare providers. Due to having a relatively small and stable population, providers often cared for multiple members of a patient’s family across several generations and were highly respected, well-known members within the rural community. Many patients reported that because of this long-standing trust, the recommendation of their healthcare provider was the most important reason why they chose to obtain diabetic eye screening. Particularly among patients who did not understand the purpose of screening, many reported obtaining regular diabetic eye screening because of the strong endorsement from their healthcare provider, which caused them to consider it an important aspect of their diabetes management.

### Individual factors

Individual factors influencing patients’ adherence with diabetic eye screening included demographic characteristics as well as emotional aspects of living with diabetes (Table 4). Patients reported several challenges related to aging, financial limitations, and the need to manage multiple health conditions in addition to their diabetes. They reported having limited time, energy, and resources to address their multiple health conditions, which led to prioritization of acute medical issues over preventive care. The cost of health insurance and healthcare services was a major concern. Many participants reported that patients with diabetes were often elderly and lived on a fixed income requiring regular financial trade-offs between their healthcare and daily living expenses. Participants reported that infrequent use of health care was a social norm among many people in the community who habitually obtained health services only when symptoms become severe rather than seeking preventive care such as diabetic eye screening.

**Table 4:** Individual factors.

Theme	Quotes
Multiple health conditions	I work with my other health issues and just, it’s kinda like a juggling match. (PT #16) I’m on oxygen. I have sleep apnea… before I go to bed at night I have… two pills that I take because I have incontinence. Otherwise if I cough, or sneeze, anything I dribble. [I’m on another pill] for my legs, because if I don’t take it, then I have a hard time… falling asleep. (PT #15)
Poverty and financial tradeoffs	[Health Insurance] got so expensive, after I got diabetes, I just couldn’t afford it. I was paying more to have health insurance than I was for my house. (PT #5) We have… a large number of people here who are just on… Medicare, Medicaid… no pensions… they’re living in a trailer and making ends meet… they’ve worked at an auto factory in Janesville… and retired here to their hunting camp. (PCP #4)
Limited health literacy	I do look up [health information] online all of the time… because I only have an 8th grade education, which is an equivalent now of a 5th grade education. When my son was in 5th grade, that was above me and I had to learn with him. (PT #11)
Infrequent use of health care	[Some patients] come in and now, and they have a very, very poor prognosis and they are terminal, because [they] literally haven’t seen a doctor in 20 or 30 years… When I look at their chart… I see that… they almost never come in. (PCP #3)
Burden of diabetes management	[Diabetes management] is an everyday thing. It isn’t just doing it once a week or once a month. It’s every day. (PT #12) I tried to diet, but dieting hasn’t come very easy. I dieted for about a month and then of course I gave up because everybody around me was not. (PT #18)
Negative self-perception	Sometimes I’m naughty. I take something, I eat it. And then I [have] too much sugar, then my count goes up. (PT #15) I know what you’re going to tell me… I know what my sugars are, I know that I’m bad, I know that I’m supposed to do a lot of things that I don’t do. You know, I don’t know what else to tell ya.’ I’m just bad. (PT #8)
Anxiety related to diabetes complications	[Diabetic eye screening is] one of those things I am very cautious about because I need my eyes and it scares me. (PT #20) Now, I heard [that with] diabetes you can go blind, that’s actually kind of scary to me. That would be pretty horrible, being blind. (PT #7)

Furthermore, some patients expressed a belief that their limited educational background (60% of patients had completed high school or fewer grade levels) negatively affected their health literacy and limited their ability to understand and/or follow diabetes management guidelines such as for diabetic eye screening. Some patients reported frustration with misunderstanding how to manage their diabetes, especially upon initial diagnosis, and patients often reported that the diabetes education they received was sometimes insufficient to help them make informed choices. For example, several patients with over 10 years’ experience living with diabetes reported that they had never received a detailed explanation regarding how diabetes can cause blindness and did not know that diabetic retinopathy treatments are highly effective for preventing blindness when the disease is detected early. Many patients reported that this type of information would further strengthen their healthcare provider’s recommendation and help motivate them to obtain diabetic eye screening.

Patients also discussed the emotional impact of living with diabetes including the burden of diabetic lifestyle changes, negative self-perception related to difficulty managing their diabetes, and anxiety related to the fear of diabetes complications including vision loss. Many patients reported a lack of self-efficacy in following diabetes management guidelines (“I gave up…” Patient #18) and subsequently labelled themselves as being “bad” or “naughty” for non-adherent behaviors. Furthermore, anxiety regarding the potential for diabetes complications such as blindness sometimes motivated patients to obtain diabetic eye screening. Other patients, however, avoided diabetic eye screening because they feared receiving bad news regarding their eye health, especially if they believed that diabetes complications were inevitable, treatments for diabetic eye disease were ineffective or that tighter glucose control was the only treatment available for diabetic eye disease. Negative feelings and self-identification discouraged some patients from obtaining diabetic eye screening by reinforcing fatalistic beliefs and a lack of confidence in their ability to prevent diabetes complications such as vision loss from diabetic eye disease.

## Discussion

Our study characterized environmental, social and individual factors influencing rural patients’ adherence with diabetic eye screening guidelines. Major themes included limited access to and infrequent use of healthcare, long travel distances to obtain care, poverty and financial tradeoffs, trusting relationships with healthcare providers, family members’ struggles with diabetes, anxiety about diabetes complications, and the burden of diabetes management.

Unlike prior studies that have focused on gaps in patient knowledge and structural barriers, we identified several prominent individual factors influencing rural patient adherence with diabetic eye screening. Rural patients reported anxiety related to diabetes complications, negative self-perception, and low self-efficacy as reasons why they or others may choose not to adhere with yearly diabetic eye screening. Emotional aspects of living with diabetes have been described in other aspects of diabetes self-management, but have not been strongly associated with diabetic eye screening [28, 29]. Some studies reported that “denial” regarding having diabetes or “not wanting to know if eye disease is present” contributed to patient nonadherence with diabetic eye screening [30, 31]. Our qualitative data help to enrich the understanding of the emotional factors underlying these attitudes that lead to avoidance of diabetic eye screening, which appear to stem from anxiety about diabetes complications, negative self-perception, and low self-efficacy.

Based on our results, we identified interrelationships of themes across environmental, social, and individual factors based on the Ecological Model of Health [23]. Since there are few eye care providers, rural patients often must travel long distances (“70 miles” PT #9) to obtain diabetic eye screening [32]. Patients often reported challenges related to being of older age, which included the burden of managing multiple medical problems, as well as limited health literacy, financial resources to pay for health services, and transportation [17, 30, 33–35]. Consequently, patients expressed a social norm of infrequent utilization of health care, particularly for preventive services such as diabetic eye screening. However, having a stable population with long-standing relationships contributed to rural patients’ having strong trust in their health care providers [12, 28, 29]. These relationships strengthened patients’ willingness to follow their health care providers’ recommendation regarding yearly diabetic eye screening. Social factors, such as painful experiences of witnessing family members’ struggle with diabetes complications, motivated some patients to obtain diabetic eye screening to protect their eyesight, but discouraged other patients from obtaining screening due to their fear of receiving bad news about their eye health. Stressful interpersonal relationships have been reported to negatively impact patients’ diabetes self-management abilities [36, 37]. Many patients reported feeling surveilled and judged negatively by their family and friends regarding their struggle with diabetes management, which reduced their self-esteem and self-efficacy for following guidelines in obtaining preventive care such as for diabetic eye screening.

Many studies recommend patient education interventions to increase adherence with diabetic eye screening, but current patient education efforts fail to adequately address environmental, social, and individual factors [9–12]. These factors include limited access to eye care providers as well as financial, time, and transportation constraints that lead patients to prioritize visits to other health care providers for more urgent or acute medical issues rather than for preventive eye care. This was particularly true among elderly rural patients in our study who frequently cited older age and struggling to manage multiple health conditions as challenges to following diabetic eye screening guidelines, especially for those who lacked insurance coverage. Despite a relatively high level of education and self-reported health literacy among rural patients in this study, many perceived themselves as having inadequate health literacy and knowledge about diabetic eye disease. They also endorsed multiple barriers to obtaining diabetic eye screening in their rural community. Our data suggest that current patient education falls short in addressing many factors, including anxiety related to diabetes complications, negative self-perception, and low self-efficacy, that limit diabetic eye screening rates in rural communities. In addition, strong healthcare provider endorsement of diabetic eye screening may be highly influential in leveraging rural patients’ trust in their healthcare providers. Social and anthropologic reasons underlying healthcare access and acceptance are likely to be critical components for informing future context-specific interventions to increase diabetic eye screening and reduce healthcare disparities, for example through the Social Behavior Change Communication (SBCC) model [38–40].

Furthermore, innovative technological strategies such as teleophthalmology may complement patient education in improving patient adherence by directly addressing environmental barriers to diabetic eye screening in rural populations such as less access and greater travel distances to obtain eye care [6–8, 41–43]. Similar barriers are present in rural communities within low and medium income countries where specialists are concentrated in urban areas [44–46]. Telemedicine has been used successfully to reach elderly diabetes patients in underserved areas of both developing and industrialized countries [44, 46, 47]. Teleophthalmology is an evidence-based form of telemedicine where a retinal camera is used for diabetic eye screening that can effectively increase screening rates in rural communities [33, 41, 48–50]. By making teleophthalmology available in primary care clinics, patients can conveniently obtain screening at low-cost when seeing their primary care provider for diabetes and other chronic disease management. However, rural communities in the U.S. are largely served by multi-payer health systems, which are less likely to encourage preventative services or to have insurance reimbursement for teleophthalmology [48, 51]. Furthermore, U.S. rural health clinics and federally-qualified health centers, where the need for access to low-cost specialty care is greatest, are currently unable to receive federal reimbursement for telemedicine services. Policies to improve reimbursement for teleophthalmology may allow for further expansion of these programs and increase access to preventive eye care in rural communities.

Limitations of our study include that all patients had some experience with diabetic eye screening. Patients who are not adherent with diabetic eye screening guidelines may have been less likely to participate and may experience different barriers. However, prior studies have shown that adherent and non-adherent patients experience similar barriers [30]. Most patients in this study self-reported high levels of health literacy (85%), which was greater than that of rural adults in a similar study (70.9%) [52]. All patients were white, native English speakers, which was representative of this rural population. Further studies among patients from other ethnic groups and non-native English speakers are needed to assess the generalizability of these findings to these populations. In addition, qualitative studies evaluating barriers to other forms of health maintenance screening (e.g. colon cancer and mammography) in rural patient populations may identify similar themes as those found in our study and be amenable to thematic categorization using the Ecological Model of Health [23].

## Conclusions

Significant barriers exist for rural patients that affect their ability to adhere with yearly diabetic eye screening. Many studies emphasize patient education to increase adherence, but current strategies fail to address major environmental, social, and individual barriers. Addressing these factors, leveraging patient trust in their healthcare providers, and strategies targeted specifically to environmental barriers such as long travel distances (e.g. teleophthalmology) may fill crucial gaps in diabetic eye screening in rural communities.

## Supporting information

S1 Text Patient Interview Guide. This is the S1 Patient Interview Guide.
(PDF)

S2 Text Primary Care Provider Interview Guide. This is the S2 Primary Care Provider Interview Guide.
(PDF)
